# Effects of Anæmia on the Eye

**Published:** 1892-01-09

**Authors:** 


### THE PRACTITIONER'S MIRROR AND RETROSPECT.

[*The Editor will be glad to receive offers of co-operation and contributions from members of the profession. All letters should be addressed to* THE EDITOR, THE LODGE, PORCHESTER SQUARE, LONDON, W.]

### MEDICINE.

### EFFECTS OF ANÆMIA ON THE EYE.

Anæmia affects the vision in all degrees, from a slight feeling of fatigue in the act of accomodation to actual and permanent blindness. It is a common thing in ophthalmic hospitals to have patients applying for impairment of vision for near things; they cannot sew or read without fatigue, especially after the lights are lit. One naturally at once thinks of hypermetropia, but the use of convex glasses does not improve the acuity of vision, the opthalmoscope shows no definite change in the fundus or the media, then one may say it is probably ciliary spasm with hypermetropia. Retinoscopy soon clears this up, and may actually reveal the refraction to be emmetropic; it therefore cannot be accounted for in this way. The appearance and history of the patient will reveal more, the patient is probably seen to be pale, she may be chlorotic, or have that characteristic appearance that a woman who has suckled too long exhibits, and further questioning brings out these facts, and examination shows the usual symptoms of general anæmia. It is in these cases that the results of treatment are brilliant. Take away the cause and treat the anæmia without any further waste of time, and the patient rapidly recovers her sight. We have even known a lady travel to England from the Cape because "her sight was failing," and moreover got worse during the voyage, and she brought the cause with her, viz., her baby. She was emmetropic, her fundi shewed no particular signs, the veins were a little lighter than usual in comparison with the arteries. She was recommended to wean her baby, take iron in the form of Bland's Pills, and keep her mind at ease. She wrote two months' afterwards to say she was quite well, and her vision as good as it had ever been. We quote this case especially, because her refraction was practically the normal standard, and up to the time her sight began to fail had been in perfect health.

In progressive anæmia the vision is not much affected; hæmorrhages, however, occur in the retina, and if these be at all central they will interfere with vision, which will improve again when they are absorbed after a few weeks. The ophthalmoscopic signs are these hæmorrhages, with the optic disc generally normal, though sometimes its edge is blurred, or both nerve and retina affected, neuro-retinitis. Other appearances have been described, but these are the commonest. In the anæmia of purpura, hæmorrhages generally are found in the retina, and these cause obstruction to vision. According to their position as regards the macula lutea, if actually on this spot, the central vision will be much affected.

But there are much graver effects on the eye from anæmia. An acute hæmorrhage, from a wound from venæsection, from the uterus post partum, and more especially from the stomach, may end in complete blindness. This is generally after a large hæmorrhage, but it may be after a series of small ones. Again, the effect on vision may be only slight, even in large hæmorrhages, or the loss of sight may be temporary, recovery taking place after a few days. Again, the blindness may follow immediately in the wake of the hæmorrhage, or may not come on till three or four days afterwards. In these cases both eyes are generally affected, showing it is due to the effect on the general visual system, but occasionally only one eye is affected, when we may conjecture that some local hæmorrhage has occurred around the optic nerve of that side. Examination of the eyes shows dilatation of the pupils which do not react to light. The ophthalmoscope shows general pallor of the retina, with a pale disc, the arteries small (atrophied), and the veins broad, from being flattened out, the blood not fully dilating them, when atrophy of the optic nerve exists, the veins too will be smaller after a time.

It is important after hæmorrhages to think of the patient's eyes. Old-fashioned monthly nurses even, know it is wrong to let the puerperal patient read for some days after the pregnancy has terminated, and the same applies to all cases in which large hæmorrhages have occurred.

Dr. Ransome and Dr. Mules state that the retinal hæmorrhages commence when the percentage of red corpuscles has dropped to 32, or thereabouts; but Dr. S. Mackenzie thinks they cannot drop below 50 per cent. without serious risk of retinal hæmorrhage, and considers it rare, as a consequence of amæmia alone, when the portion of red corpuscles is above 50 per cent.

Anæmia, therefore, may present no eye symptoms, or simply defect of vision, loss of visual acuity, or temporary or permanent blindness. There may be no ophthalmoscopic symptoms, or there may be optic atrophy, neuro-retinitis, retinal hæmorrhages.

If the perimeter be employed, scotoma will be found on the chart corresponding to the sites of the retinal hæmorrhages. When the optic nerve is affected, the visual field will show much contraction for colours, just the same as when no anæmia is present.

